# Effect of an Office-Based Surgical Safety System on Patient Outcomes

**Published:** 2012-12-25

**Authors:** Noah M. Rosenberg, Richard D. Urman, Sean Gallagher, John Stenglein, Xiaoxia Liu, Fred E. Shapiro

**Affiliations:** ^a^University of Massachusetts Memorial Medical Center, Worcester; ^b^Harvard Medical School, Brigham and Women's Hospital; ^c^Tufts University School of Medicine; ^d^Harvard Medical School, Beth Israel Deaconess Medical Center, Boston, Mass

## Abstract

**Objective:** To implement a customizable checklist in an interdisciplinary, team-based plastic surgery setting to reduce surgical complications. **Methods:** We examined the effects on patient outcomes and documentation of a customizable, office-based surgical safety checklist. On the basis of the World Health Organization Surgical Safety Checklist, we developed a 28-element, perioperative checklist for use in the office-based surgical setting. The checklist was implemented in an office-based plastic surgery practice with an already high standard of care. We recorded baseline, prechecklist rates for each checklist item and postoperative adverse outcomes via a retrospective chart review of 219 cases. After an education program and 30-day run-in period, a prospective, post–checklist implementation chart review was initiated (n = 184), with outcome data compared to the baseline. **Results:** The total number of complications per 100 patients decreased from 15.1 to 2.72 after checklist implementation (*P* < .0001), for an absolute risk reduction of 12.4. The proportion of patients with one or more complications decreased from 11.9% to 2.72% (*P* = .0006). Site and side marking increased from 69.9% prechecklist to 97.8% (*P* < .0001). Medical optimization increased from 90.9% to 99.5% (*P* < .0001). Emergency medical services (EMS) policy confirmation, case-specific equipment availability, anticipation of estimated blood loss, and verbal confirmation of local anesthetic toxicity precautions increased from 0% to 90.0% (*P* < .0001), 92.4% (*P* < .0001), 82.1% (*P* < .0001), and 91.3% (*P* < .0001), respectively. Assessment of patient satisfaction increased from 57.1% to 90.8% (*P* < .0001). **Conclusions:** Implementation of a customizable checklist was associated with a reduction in surgical complications in an office-based plastic surgery practice with an already high standard of care.

In recent years, the economic pressures of medicine have incited a paradigm shift in health care delivery, such that surgical procedures are moving from the hospital to the office-based setting.^1^ Safety in the office-based setting has been extensively studied.^2^^-^5 Often called the “Wild West of health care,” office-based practices are not uniformly regulated, and office-based procedures continue to increase at a rapid rate, with an estimated more than 10 million procedures performed in 2010.

Recent studies found that a comprehensive checklist used in an interdisciplinary, team-based setting resulted in reduced surgical complications and cost savings.^6^^-^8 In particular, the SURPASS Trial showed that checklist implementation in hospitals reduced complications from 27.3 to 16.7 per 100 patients.^6^ In addition, the Safe Surgery Saves Lives study group at the WHO found that checklist use in 8 hospitals around the world was associated with a reduction in major complications from 11.0% to 7.0%.^9^

Many checklist trials have been performed in hospitals, but the office-based setting remains relatively unstudied. A recent editorial pointed out that according to the Agency for Healthcare Research and Quality, only 10% of patient safety studies have been performed in outpatient settings; it concluded that the office-based patient safety is often “fragmented and disorganized and lacking in clear leadership,” and the authors called for “creating a culture of safety.”^1^ This mounting concern about safety in the office-based setting led us to the development of an office-based surgical safety system that follows the office-based surgical pathway from taking a history and physical to assessing postoperative patient satisfaction. We evaluated the effect of this system on documentation, safety measures, and patient outcomes in a plastic surgery practice with a high baseline standard of care.

## METHODS

**Figure 1:**
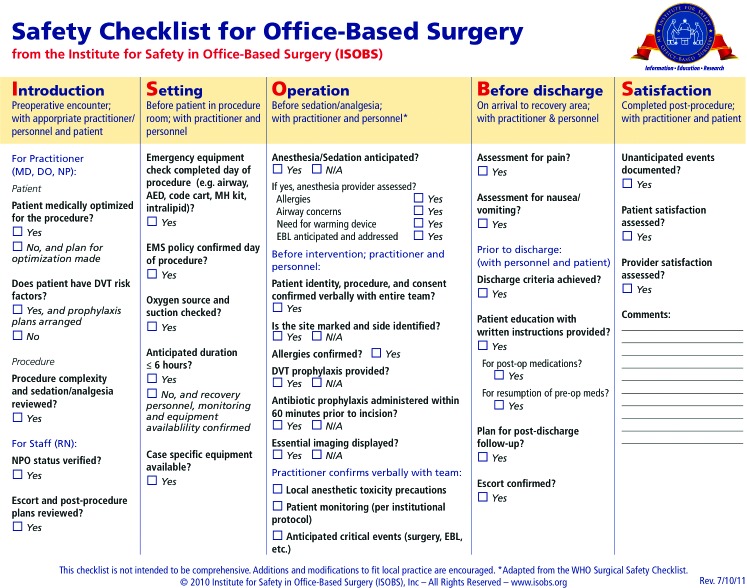
Office-based surgical safety checklist.

The development, validation, and effect on outcomes of the World Health Organization Surgical Safety Checklist have been described elsewhere.^6^^,^^10^ On the basis of the World Health Organization Surgical Safety Checklist, we developed a 28-element, perioperative checklist template for use in the office-based surgical setting (Fig 1). The checklist is divided into sections that correspond to the stages of care in the office-based surgical pathway (Introduction, Setting, Operation, Before discharge, and Satisfaction). The checklist is inherently multidisciplinary, as the surgeon, anesthesiologist, and nurse are all responsible for completion of specific checklist items. Items on the checklist include, but are not limited to, ensuring site and side marking, medical optimization of the patient, and assessment of patient satisfaction with the procedure (Fig 1).

The effects of the checklist on patient outcomes and documentation were studied in a single-center, prospective study comparing outcomes before and after implementation of the checklist from February 2010 to March 2012. The checklist was implemented in a plastic surgery practice with an already high standard of care in a suburb of a major metropolitan city. Before implementation of the checklist, the practice utilized numerous protocols for various parts of the surgical pathway, including marking the operative side and quantitative assessment of readiness for discharge. A focus group of surgeons, anesthesiologists, and nurses from the practice provided input that allowed customization of the checklist to the practice. The amount of time required to implement the checklist was estimated at 2 to 3 months, including focus group customization, an education program, and a 30-day run-in period. The baseline measurement period was 10 months. During this period, complication and prechecklist rates of completion for each checklist item were determined via a retrospective chart review of 219 contiguous cases. No patients were excluded from this group. After implementation of the checklist during a 2-month period, a postchecklist assessment of complication rates and item-specific checklist completion rates were determined for 184 contiguous cases. Two cases were excluded from statistical analysis because the checklist was not utilized, yielding a compliance rate of 99.0%.

The study was reviewed and approved by the institutional review board of Beth Israel Deaconess Medical Center. Because this was an observational study in which the effect of a quality-improvement intervention was assessed with the use of outcome measures that are already routinely collected, the board determined that formal review and informed consent were not required.

All recorded complications were classified into 12 categories (seroma formation, uncontrolled pain, bleeding, rash, nausea/vomiting, emergency medical services activation, hypotension, infection, neurologic deficit, hyperglycemia, death, and reoperation). The number of each complication per 100 patients and the proportion of patients with one or more complications were reported. The Pearson chi-square test or the Fisher exact test were used to compare prechecklist and postchecklist implementation where appropriate. Absolute risk reduction was calculated to quantify the checklist's effect on complication rates. Using an alpha level of .05, a 2-sided likelihood ratio test yielded a power of .976. SAS 9.3 was used for statistical analysis.

## RESULTS

The preimplementation cohort consisted of 212 patients, of whom 3.3% underwent more than 1 procedure. The total number of surgical procedures was 219 (Table 1). In the postimplementation cohort, 180 patients underwent 184 procedures; 2.2% underwent more than 1 procedure (Table 1). A mean of 87% of items were completed per checklist.

After implementation of the checklist, the total number of complications decreased from 15.1 complications per 100 patients to 2.72 per 100 patients (*P* < .0001), corresponding to an absolute reduction of 12.4 complications and to an uncorrected rate ratio of 0.180 (Table 1). The proportion of patients with 1 or more complications was 11.4% in the preimplementation period and 2.72% in the postimplementation period (*P* = .0006).

Without reaching statistical significance, the proportion of patients who had temporary disability and the proportion of patients requiring a second surgical procedure decreased 0.45 percentage points (Table 2). In addition, activation of emergency medical services and infection each decreased 0.91 percentage points (Table 2). There were no deaths in either cohort (Table 2).

**Figure 2:**
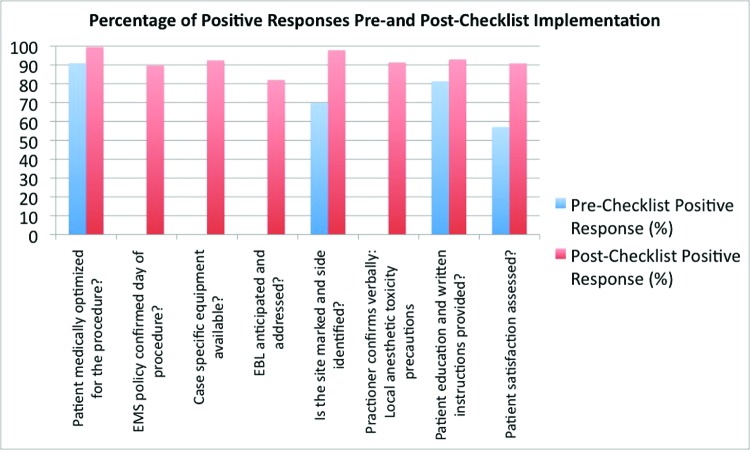
Percentage of positive responses pre-implementation and post-implementation (all P < 0.05).

In terms of effect on safety practices, site and side marking increased significantly from 69.9% preimplementation to 97.8% postimplementation (*P* < .0001) for an uncorrected rate ratio of 1.40 (Fig 2). Medical optimization of patients, or ensuring that they had a history and physical performed within 48 hours of surgery, increased from 90.9% to 99.5% (*P* < .0001) for an uncorrected rate ratio of 1.10 (Fig 2). Assessment of patient satisfaction increased significantly from 57.1% to 90.8% (*P* < .0001) for an uncorrected rate ratio of 1.59 (Fig 2). EMS policy confirmation, case-specific equipment availability, anticipation of estimated blood loss, and verbal confirmation of local anesthetic toxicity precautions increased from 0% to 90.0% (*P* < .0001), 92.4% (*P* < .0001), 82.1% (*P* < .0001), and 91.3% (*P* < .0001), respectively (Fig 2).

## DISCUSSION

In this single-center study, implementation of the office-based surgical safety checklist in a plastic surgery practice with a high baseline standard of care was associated with a reduction in postoperative complication rate from 15.1 complications per 100 patients before implementation to 2.72 per 100 patients afterward. Improved outcomes after checklist implementation may be explained by a number of mechanisms. The checklist is designed to incorporate existing protocols to provide a customizable framework for the office-based surgical setting, minimize disruption of care from one stage of the procedure to the next, and, perhaps most importantly, promote interdisciplinary communication and collaboration.

Specific items on the checklist may directly prevent adverse events. For example, medical optimization of patients prior to surgery could help avoid perioperative and postoperative complications. In addition, site and side marking, which significantly increased, could potentially avoid countless “never events.” It should be noted, however, that checklist use is not associated with a statistically significant reduction in specific types of complications, only the total number of complications. Therefore, it would not be statistically rigorous to speculate on how the checklist reduces seroma formation, for example, because a larger study population would be necessary to obtain statistical significance. The checklist may also improve outcomes by generally improving documentation, as evidenced by the number of checklist items that increased from 0% preimplementation to significantly higher rates of postimplementation. Furthermore, the checklist may enhance outcomes by improving attitudes toward quality improvement, communication, teamwork, and patient safety.

The improvements in outcomes that we observed validate the results that were achieved with the use of surgical safety checklist of the World Health Organization and the SURPASS Trial.^6^^,^^10^ Indeed, the absolute risk reduction in complications were somewhat similar, at 12.6 for this study and 10.6 for the SURPASS Trial. The most obvious difference between those studies and this study is the difference between hospital- and office-based surgery. In addition, those studies featured checklists that were meant to be comprehensive without the possibility for inclusion or exclusion of a given item based on the specific center's needs. This study, however, featured a fully customizable checklist that can be tailored to the specific practice, a strength, which lends itself to the diversity of practices in the office-based setting.

This study has several limitations. First, because it had preimplementation and postimplementation phases, any effect of the intervention may have been influenced by other changes that occurred over time or by differences in case type. A randomized study design, however, is not feasible for this type of study because office personnel using the checklist for one case will continue to consciously or subconsciously use it for a case not randomized to the checklist. In an effort to minimize this effect, contiguous cases were examined in the preimplementation and postimplementation cohorts. In addition, to further minimize temporal effects, no other changes in office policy or surgical care were implemented during the duration of the study, making it unlikely that the decrease in complications was attributable to factors other than the checklist.

Second, the preimplementation and postimplementation cohorts were not compared in terms of demographic data and patient characteristics to ensure intergroup homogeneity. Again, however, study of contiguous cases was meant to minimize the effects of confounders and selection bias. On a related note, the use of a single-center design limited the study to a specific patient population, though confirmation of our results by the SURPASS Trial and others put to rest any question of generalizability. The sample size may also raise questions of generalizability, but the power calculation of 0.976 far exceeded the generally accepted level of 0.8.

Third, documentation by physicians has proven to be subject to underreporting,^11^^,^^12^ which may have affected complication rates, though one might logically conclude that the preimplementation and postimplementation cohorts would be equally affected.

Fourth, the documentation of complications was limited to the duration of the procedure or the days immediately following the procedure. Data on complications occurring thereafter were not collected.

Finally, the checklist was not fully complied with, or completed by health care providers. Compliance was high at 99.0%, but a mean of 87% of items per checklist were completed (which is, nevertheless, a greater rate of completion than was found in the SURPASS Trial).^6^ Importantly, suboptimal compliance and completion may have underestimated the effect of the checklist.

The implementation of this checklist requires a substantial input of time and effort on the part of every member of the office-based practice. Because it is customizable, the checklist invites input from a multidisciplinary team. By providing a framework for the safe, ideal office-based surgical procedure, the checklist reveals deficiencies and triggers direct and indirect improvements in the provision of safe care. This study shows that the use of a customizable, office-based surgical safety checklist is associated with improvements in safety measures, documentation, and a reduction in complications in a practice with an already high baseline standard of care.

## Figures and Tables

**Table 1:** Cohort complication rates

	Preimplementation	Postimplementation	*P*
Number of procedures	219	184	
Number of patients	212	180	
Total number of complications	33	5	
Complication rate per 100 patients	15.1	2.72	<.0001
Proportion of patients with 1 or more complications	11.40%	2.72%	.0006

**Table 2:** Complication types and rates

Preimplementation Number (% total)	Complication	Postimplementation Number (% Total)	*P*
4 (1.82)	Seroma formation	0	.1288
8 (3.65)	Poorly controlled pain	3 (1.49)	.2145
3 (1.37)	Bleeding	0	.2538
5 (2.28)	Rash	0	.0657
5 (2.28)	Nausea/vomiting	1 (0.495)	.2259
2 (0.913)	Activation EMS	0	.5025
1 (0.457)	Hypotension	1 (0.495)	1
2 (0.913)	Infection	0	.5025
1 (0.457)	Neurologic deficit (BUE weakness)	0	1
1 (0.457)	Hyperglycemia	0	1
0	Death	0	N/A
1 (0.456)	Reoperation	0	1
33	Total	5	<.0001

BUE indicates bilateral upper extremity.
